# The International Medical Congress at Rome

**Published:** 1894-04-14

**Authors:** 


### The International Medical Congress at Rome.

(By our own Correspondent.)

Professor Michael Foster on "Organisation of Science."

Professor Michael Foster delivered an address on "Organisation in Science as an Aid to Scientific Results." He pointed out that the salient points of an organised creature were differentiation on the one hand, and on the other integration. The members of its body all work together for the common good. In the body politic the same thing occurred. Integration was carried out by the unwritten customs or by the enacted laws and regulations. The workers in science all the world over formed such a body. Differentiation had gone far. There was a time when one man could push forward by himself several sciences. Now each one had to be content to devote himself to a single science or even to a small portion of that science. Differentiation in this respect must go still further. Regarding integration of science, it seemed that the great International Congress, at which so many nations were gathered together, especially a Congress of Medicine, which is the mother of all the sciences, might be a fitting body, and that this eternal City of Rome within the bosom of the seven hills, once the central knot at which were tied the cords of organisation, might be a fitting place for putting forward the question, if there were not difficulties which required to be overcome by integration among the workers in science. Surely it was possible for human wit to invent some ties which would bind together the workers in science for their common good. Organisation might be applied to inquiry itself and also to the circumstances affecting it, the means by which it was to be accomplished. There were the two kinds of inquiry: the individual and the combined. He did not underrate the danger of organised inquiry in selecting the wrong men. An inquirer, like a poet, was born, not made, and there might be a certain amount of tyranny in exercising the machinery of an organisation, which might destroy individual inquiry. It might interfere with the motives, which sustained such an inquiry. There was the motive of ambition, the love of fame. There was also the love of curiosity. Organisation, however, would prevent men from going over the same ground, and guide young workers who did not know where to begin. The publication of crude material appeared to Professor Foster as sewage contamination defiling the pure stream of science. He concluded by urging, that if organisation were a good thing, nations should join together and to carry it forward, and it would undoubtedly follow, as water flows downward, that good would result from it. The great initial expenditure would soon be overcome, and would be compensated for by the great advantage which would accrue to medical science if an international organisation could be brought about. Individual ambition would then give way to the love of truth, which was the only safe guide, so far as the work of science was concerned.

This address by Professor Michael Foster formed one of the principal features for the English members on the day when the work of the Congress actually began. Throughout the meetings the number of papers put down for hearing was enormous, and on most days it was impossible to carry out the intended programme. We gave the list of subjects brought forward by the English practitioners in the issue of last week. Amongst the foreign contributions were the interesting demonstrations given in the Section of Internal Medicine by Professor Baccelli, Rome, and Dr. Ebstein, Göttingen, of their respective methods of determining the force and volume of the action of the heart, a number of invalids, as well as persons in good health, being brought in to illustrate the theories of the lectures. The subject of tuberculosis in the larynx was very exhaustively dealt with in the Section of Laryngology. The official reporters, Dr. Gouguenheim, Paris, and Dr. Heryng, Warsaw, opened the discussion with a brilliant exposition of the indications and local treatment of the disease.

Signor Postemski performed two operations, radical cure of hernia and hysterectomy for the benefit of several eminent Congressists, and in the Clinico Chirurgio, Signor Durante removed a large left kidney from a woman in the presence of Sir Spencer Wells, Sir William MacCormac, and other distinguished Congressists. The operation was performed under the most rigid antiseptic precautions, chloroform administered, and wire sutures used for bringing the superficial wound together, the deep parts being brought together by silk continuous suture. There was a large cyst at the upper part of the kidney containing pus, which was emptied first by aspirator, then refilled with carbolized water, and the aperture closed before proceeding to remove the organ. A calculus, the size of a marble, was found in the hilus of the kidney, when after removal it was divided in half.

The attention of those attending the Congress was by no means confined strictly to the business of the meeting. The amusements provided were many and varied. In these the King and Queen played a most gracious part, devoting much time to the Congress. Banquets were given to each section, divided into: 1, Anatomy and Physiology; 2, General Pathology and Pathological Anatomy; 3, Internal Medicine and Pediatry; 4, Psychiatry, Neuropathology, Dermatology, and Syphilopathology; 5, Surgery and Orthopædy; 6, Laryngology, Otology, and Ophthalmology; 7, Hydrology, Climatology, Sanitary Engineering, and Hygiene; and 8, Odontology. The Minister of Public Instruction received Professor Virchow and the most distinguished foreign and native physicians. Mr. Burroughs, of the firm of Burroughs and Welcome, gave a brilliant ball to English and American guests. A reception in the Capitol was given by members of the Municipality. The King and Queen gave a garden party at the Quirinal, and the English Ambassador also held a reception. There was a gala performance at the theatre. The festivities ended by a lunch at the Baths of Caracalla. During the Congress the Pope held a special service in the Vatican. An interesting excursion was made in a military train to Tivoli, where a special fête was held for those more especially interested in military surgery and ambulance work. At the conclusion of the Congress, Signor Baccelli bid the members a formal adieu on behalf of the Government.

